# Synthesis and Bioactivity of 5-Substituted-2-furoyl Diacylhydazide Derivatives with Aliphatic Chain

**DOI:** 10.3390/ijms15058941

**Published:** 2014-05-20

**Authors:** Zining Cui, Xinghai Li, Fang Tian, Xiaojing Yan

**Affiliations:** 1 Guangdong Province Key Laboratory of Microbial Signals and Disease Control, Department of Plant Pathology, College of Natural Resources and Environment, South China Agricultural University, Guangzhou 510642, China; 2 State Key Laboratory for Biology of Plant Diseases and Insect Pests, Institute of Plant Protection, Chinese Academy of Agricultural Sciences, Beijing 100193, China; E-Mail: ftian@ippcaas.cn; 3 Department of Pesticide Science, Plant Protection College, Shenyang Agricultural University, Shenyang 110866, China; E-Mail: xinghai30@163.com

**Keywords:** diacylhydrazide, aliphatic chain, synthesis, bioactivity

## Abstract

A series of 5-substituted-2-furoyl diacylhydazide derivatives with aliphatic chain were designed and synthesized. Their structures were characterized by IR, ^1^H NMR, elemental analysis, and X-ray single crystal diffraction. The anti-tumor bioassay revealed that some title compounds exhibited promising activity against the selected cancer cell lines, especially against the human promyelocytic leukemic cells (HL-60). Their fungicidal tests indicated that most of the title compounds showed significant anti-fungal activity. The preliminary structure-activity relationship showed that the aliphatic chain length and differences in the R^2^ group had obvious effects on the anti-tumor and anti-fungal activities. The bioassay results demonstrated that the title compounds hold great promise as novel lead compounds for further drug discovery.

## 1. Introduction

Hydrazides, a group of peptide mimicking molecules with amide group and flexible conformation, have been applied in medicine since the early 1950s, when isoniazid was used as a therapeutic agent against *Mycobacterium tuberculosis* [1,2]. Later studies discovered their diverse bioactivities as antituberculous agents [3,4], human immunodeficiency virus (HIV) inhibitors [5], inhibitors of microperoxidase [6] and glycogen phosphorylase [7], and pesticides [8–10]. Hydrazides are also key intermediates, especially in the preparation of pharmaceuticals and agrochemicals. Their synthesis has attracted significant attention due to their application as scaffolds in the construction of many nitrogen containing heterocycles, such as 1,2,4-triazoles [11], 1,3,4-oxadiazoles [12,13], 1,3,4-thiadiazoles [14], 1,2,4,5-tetrazines [15], *etc*.

**Scheme 1.**
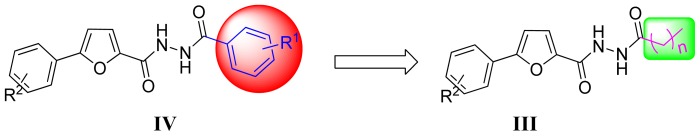
Design strategy for title compounds.

**Scheme 2.**
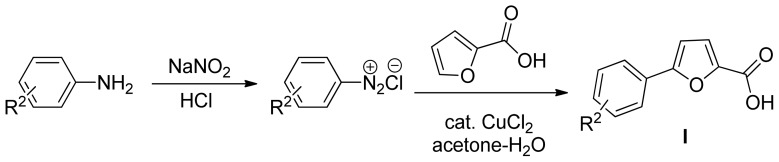

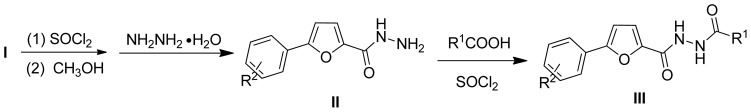
General synthesis procedure for title compounds **III. III-1-1**: R^1^ = CH_3_, R^2^ = 2-Cl; **III-2-1**: R^1^ = CH_3_, R^2^ = 3-Cl; **III-3-1**: R^1^ = CH_3_, R^2^ = 4-Cl; **III-4-1**: R^1^ = CH_3_, R^2^ = 2-F; **III-5-1**: R^1^ = CH_3_, R^2^ = 3-F; **III-6-1**: R^1^ = CH_3_, R^2^ = 4-F; **III-7-1**: R^1^ = CH_3_, R^2^ = 2,4-di-F; **III-8-1**: R^1^ = CH_3_, R^2^ = 2,6-di-F; **III-9-1**: R^1^ = CH_3_, R^2^ = 2-NO_2_; **III-10-1**: R^1^ = CH_3_, R^2^ = 3-NO_2_; **III-11-1**: R^1^ = CH_3_, R^2^ = 4-NO_2_; **III-12-1**: R^1^ = CH_3_, R^2^ = H; **III-13-1**: R^1^ = CH_3_, R^2^ = 4-CH_3_; **III-14-1**: R^1^ = CH_3_, R^2^ = 4-OCH_3_; **III-15-1**: R^1^ = CH_3_, R^2^ = 4-Br; **III-3-2**: R^1^ = CH_2_CH_3_, R^2^ = 4-Cl; **III-3-3**: R^1^ = *n*-C_3_H_7_, R^2^ = 4-Cl; **III-3-4**: R^1^ = *i*-C_3_H_7_, R^2^ = 4-Cl; **III-3-5**: R^1^ = *n*-C_5_H_11_, R^2^ = 4-Cl; **III-3-6**: R^1^ = *n*-C_6_H_13_, R^2^ = 4-Cl; **III-3-7**: R^1^ = *n*-C_7_H_15_, R^2^ = 4-Cl; **III-3-8**: R^1^ = *n*-C_8_H_17_, R^2^ = 4-Cl; **III-3-9**: R^1^ = *n*-C_9_H_19_, R^2^ = 4-Cl.

In our previous study, we found that 5-phenyl-2-furoyl diacylhydrazides containing aromatic rings (**IV**) [16–22] not only exhibited anti-tumor activity, but also fungicidal and insecticidal activity. In order to discover compounds with better bioactivities, we replaced the rigid aromatic rings (**IV**) with the flexible aliphatic chains (**III**), as shown in Scheme 1. A series of 5-phenyl-2-furoyl diacylhydrazides bearing different aliphatic chains were designed and synthesized by the route shown in Scheme 2. Their anti-tumor, anti-fungal, and insecticidal activities were evaluated. The structure-activity relationships were elucidated.

## 2. Results and Discussion

### 2.1. Synthesis and Structure Elucidation

By utilizing the method of Meerwein arylation with copper(II)-catalyzed decomposition of diazonium salts, a series of 5-substituted phenyl-2-furoic acid **I** were prepared in good yields, based on substituted anilines and furoic acid as starting reagents. The nucleophilic reaction with 2-furoic acid exhibited a high region-selectivity at the 5-position of the furan ring. Then the 5-substituted phenyl-2-furoyl hydrazides **II** were prepared as previously described [16–22], and subsequently reacted with different aliphatic acids and thionyl chloride to obtain the title compounds. The synthesis route of title compounds **III** is shown in Scheme 2.

All the structures of the title compounds were confirmed by IR, ^1^H NMR, and elemental analyses. In the IR spectra, the compounds showed absorption bands around 3300 cm^−1^, originating from the N–H stretching vibration. The strong bands around 1680 cm^−1^ could be assigned to the C=O stretching vibration. The bands around 1620 cm^−1^ were attributed to the secondary amide. Absorption bands around 1510 and 1480 cm^−1^ were attributed to the frame vibration of the phenyl and furan rings.

In the ^1^H NMR spectra, one or two sharp peaks in the range from 10.30 to 10.80 ppm were due to the presence of hydrazine. Mostly, the signals for protons on the phenyl rings appeared as multiplets in the range from 7.10 to 8.20 ppm and the signals for protons on the furan ring were split into two doublets in the range from 6.90 to 7.05 ppm. All the proton signals of the aliphatic chain appeared in the high field around 0.80 to 2.20 ppm.

**Figure 1.**
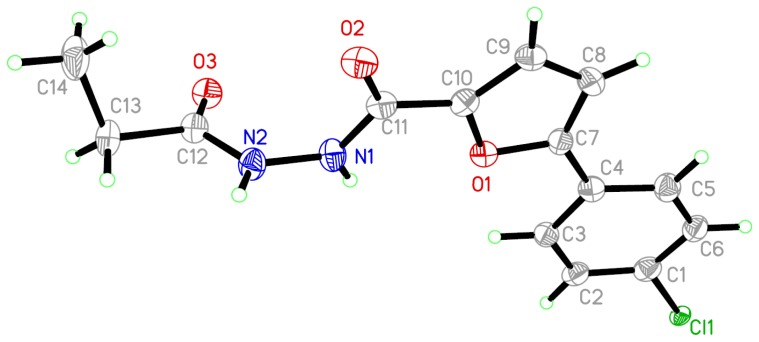
Molecular structure of compound **III-3-2**, showing 30% probability ellipsoids; H atoms are shown as small spheres of arbitrary radii.

The crystal data and structure are presented in Table 1 and Figure 1, and allowed a perspective view of compound **III-3-2**. Some important bond lengths, angels, and torsion angles of compound **III-3-2** are given in Table 2. It can be seen from the X-ray single crystal analysis of **III-3-2** that the distance of single bonds C11–N1 and C12–N2 (1.355(6) and 1.343(6) Å) were equal to the C–N double bond (1.35 Å), the single bonds C4–C7 and C10–C11 (1.455(6) and 1.465(6) Å) were shorter than the standard C–C single bond (1.54 Å), but longer than the C–C double bond (1.34 Å). N1–N2 (1.396(5) Å) single bonds was shorter than the standard N–N single bond (1.45 Å), but longer than N–N double bond (1.25 Å). These results clearly indicated that the p orbital of the N atoms conjugated with the π molecular orbital and formed the delocalized π-bonds with the conjoint furan and benzene ring. However, unexpectedly, the p orbitals of N1 and N2 seemed not to be conjugated with the π molecular orbital of the C11–O2 and C12–O3 double bonds, which was explained by the bond length of C11–O2 and C12–O3 (1.238(5) and 1.218(5) Å) that followed the normal range for C–O double bond lengths (1.19–1.23 Å).

In the crystal structure, C(1), C(2), C(3), C(4), C(5), and C(6) formed a plane with a mean deviation of 0.0153 Å, defined as plane I; C(7), C(8), C(9), C(10), and O(1) formed a plane with a mean deviation of 0.0150 Å, defined as plane II; O(2), C(11), N(1), and N(2) formed a plane with a mean deviation of 0.0047 Å, defined as plane III; O(3), C(12), C(13), and C(14) were not coplanar, which were defined factitiously as plane IV with a mean deviation of 0.3708 Å (Figure 1 and Table 3). Planes II, III, IV formed a dihedral angle with plane I of 10.0°, 12.4°, and 56.9°. Planes III and IV formed a dihedral angle with plane II of 6.8° and 47.7°, and the dihedral angle between planes III and IV was 50.6°. The related data is summarized in Table 3.

### 2.2. Bioassay

#### 2.2.1. Insecticidal Activity

Considering that our previous aromatic diacylhydrazide compounds displayed good insecticidal activities against dipterous and lepidopterous insects, *Plutella xylostella*, *Mythimna separata*, and *Culex pipiens pallens* were chosen to evaluate whether the aliphatic compounds retained the insecticidal activity. The results in Table 4 show that compounds **III** had moderate to poor insecticidal properties. The best one was compound **III-8-1**, with activities of 60%, 50%, and 60% against *P. xylostella*, *M. separata*, and *C. pipiens pallens* at 200, 200, and 10 mg·L^−1^, respectively. Comparison of the activity between the title compounds and the aromatic diacylhydrazide compound **IV**-**1** from our previous study [16–22] revealed that the replacement of the rigid aromatic rings (**IV**) by the flexible aliphatic chains (**III**) had a negative effect on the insecticidal activity.

#### 2.2.2. Fungicidal Activity

The *in vivo* anti-fungal activities of the title compounds against *Fusarium oxysporum*, *Corynespora cassiicola*, *Botrytis cinerea*, and *Rhizoctonia solanii* are listed in Table 5. Most of the title compounds showed moderate to good activity against *B. cinerea* and *R. solanii* at 500 μg·mL^−1^ *in vivo*. For instance, the inhibitory rates of **III-11-1** and **III-3-1** against *B. cinerea* were 92.52% ± 2.71% and 84.23% ± 2.43%, which was equal to the positive control 40% pyrimethanil SC (89.57% ± 2.15%). The inhibitory rates of **III-3-1** and **III-9-1** against *R. solanii* were 93.43% ± 1.63% and 88.62% ± 1.62%, which was the same as the control level of 3% validamycin AS (92.21% ± 2.41%). The aliphatic chain length showed significant effects on the fungicidal activities against *B. cinerea* and *R. solanii.* In general, the shorter the chain, the better the corresponding activity was. The structure and position of R^2^ also affected the fungicidal activity against *R. solanii*. When R^2^ was NO_2_ and Cl at the *ortho* or *para* position, such as in **III-3-1**, **III-1-1**, **III-9-1**, and **III-11-1**, a higher fungicidal activity was observed. The results showed that *meta* substitution on the phenyl ring decreased the activity, and the electron-donating groups at the *para* position significantly decreased the activity. All the compounds showed moderate to poor activity against *F. oxysporum* and *C. cassiicola*. Compared with aromatic diacylhydrazide **IV**-**1**, the aliphatic derivatives showed improved and increased fungicidal activity.

#### 2.2.3. Anti-Tumor Activity

The *in vitro* anti-tumor activity of the title compounds against four cancer cell lines is listed in Table 6. Most of the title compounds showed inhibition activity against the tested cancer cell lines. The activity of **III-3-1** (IC_50_ = 21.6 μM), **III-1-1** (IC_50_ = 25.3 μM) and **III-8-1** (IC_50_ = 25.9 μM) was better than that of doxorubicin (IC_50_ = 35.6 μM) against human promyelocytic leukemic cells (HL-60). The activity of **III-3-2** (IC_50_ = 35.8 μM) was similar to that of doxorubicin (IC_50_ = 35.6 μM) against HL-60. **III-8-1** exhibited good inhibition activity against solid human gastric carcinoma cells (BGC-823), with an activity (IC_50_ = 10.8 μM) that was close to that of doxorubicin (IC_50_ = 10.2 μM). **III-3-1** and **III-8-1** also had good activity (IC_50_ = 15.2 and 18.9 μM) against human hepatocellular carcinoma cells (Bel-7402). However, the activity of title compounds against human nasopharyngeal carcinoma cells (KB) was poor.

The preliminary structure-activity relationship showed that the aliphatic chain length had a significant impact on the anti-tumor activity against HL-60. Generally, the shorter the chain, the better the corresponding activity was. The structure and position of R^2^ also affected the anti-tumor activity against HL-60. When R^2^ was Cl and F at the *ortho* or *para* position, such as in **III-3-1**, **III-1-1**, and **III-8-1**, a higher anti-tumor activity was observed. The anti-tumor activity of the title compounds was much better than that of the aromatic diacylhydrazides.

## 3. Experimental Section

### 3.1. General Information

Melting points were determined with a Cole-Parmer melting point apparatus (Cole-Parmer, Vernon Hills, IL, USA) (thermometer was uncorrected). IR spectra were recorded on a NEXUS-470 FTIR spectrometer (International Equipment Trading Ltd., Vernon Hills, IL, USA) with KBr pellets. ^1^H NMR spectra were recorded with a Bruker DPX300 instrument (Bruker, Billerica, MA, USA) and tetramethylsilane was used as an internal standard. Analytical thin-layer chromatography (TLC) was carried out on precoated plates (silica gel 60 F254) and spots were visualized under ultraviolet light. Elemental analyses (C, H and N) were carried out with a Flash EA 1112 elemental analyzer (Thermo Finnigan, Bremen, Germany). Mass spectra were measured on a Bruker ESQUIRE-LC spectrometer (Bruker, Fallanden, Switzerland). The X-ray crystal diffraction (Bruker, Fallanden, Switzerland) data were collected with a Rigaku Saturn diffractometer at 294(2) K and the crystal structures were calculated using the SHELXL program package and refined by full-matrix least squares procedures at Nankai University (Tianjin, China).

### 3.2. Synthetic Procedures

#### 3.2.1. General Synthetic Procedure for the Key Intermediates

Preparation of different 5-substituted phenyl-2-furoic acid **I** was performed according to the method described in references [16–22] and the 5-substituted phenyl-2-furoyl hydrazides **II** was performed as previously described [16–23].

#### 3.2.2. General Synthetic Procedure for the Title Compounds **III**

A mixture of aliphatic acid (0.05 mol) and thionyl chloride (0.15 mol) was refluxed in anhydrous benzene for 3 h. The excess thionyl chloride and the solvent were distilled off and the residue was dissolved in anhydrous dichloromethane. The resultant solution was added to 5-substituted phenyl-2-furoyl hydrazides **II**. The mixture was stirred and refluxed for 5 h. After cooling, the solid was filtered and recrystallized from ethyl acetate to obtain the title compounds. All the title compounds were solid. Their structures were confirmed by ^1^H NMR, IR, MS, and elemental analysis.

***N*-Acetyl-*N*′-[5-(2′-cholorophenyl)-2-furoyl]hydrazine (III-1-1).** White solid: yield 79.4%, m.p. 152–153 °C. IR (KBr) *ν*_max_: 3416.8, 3223.2, 2987.5, 1641.5, 1534.2, 1473.3, 1265.7, 1185.1, 1034.7 cm^−1. 1^H NMR (300 MHz, DMSO-*d_6_*) δ: 2.16 (s, 3H, CH_3_), 7.33 (d, *J* = 3.69 Hz, 1H, FuH), 7.39 (d, *J* = 3.69 Hz, 1H, FuH), 7.41–7.54 (m, 2H, 2ArH–Fu), 7.61–7.64 (m, 1H, ArH–Fu), 8.23 (m, 1H, ArH–Fu), 10.43 (s, 1H, NH), 10.61 (s, 1H, NH). MS/ESI: *m*/*e* (%) 301.1 [M + Na]^+^. Anal. Calcd. (%) for C_13_H_11_ClN_2_O_3_: C, 56.03; H, 3.98; N, 10.05. Found: C, 56.22; H, 3.81; N, 10.20.

***N*-Acetyl-*N*′-[5-(3′-cholorophenyl)-2-furoyl]hydrazine (III-2-1).** White solid: yield 73.6%, m.p. 136–137 °C. IR (KBr) *ν*_max_: 3412.5, 3243.1, 3023.3, 1642.2, 1524.1, 1462.4, 1327.2, 1263.3, 1182.4, 1063.1 cm^−1. 1^H NMR (300 MHz, DMSO-*d_6_*) δ: 2.16 (s, 3H, CH_3_), 7.30 (d, *J* = 3.63 Hz, 1H, FuH), 7.34 (d, *J* = 3.63 Hz, 1H, FuH), 7.47–7.52 (m, 2H, ArH–Fu), 7.93–7.96 (m, 1H, ArH–Fu), 8.15 (m, 1H, ArH–Fu), 10.46 (s, 1H, NH), 10.76 (s, 1H, NH). MS/ESI: *m*/*e* (%) 279.1 [M + H]^+^. Anal. Calcd. (%) for C_13_H_11_ClN_2_O_3_: C, 56.03; H, 3.98; N, 10.05. Found: C, 55.89; H, 4.18; N, 9.87.

***N*-Acetyl-*N*′-[5-(4′-cholorophenyl)-2-furoyl]hydrazine (III-3-1).** White solid: yield 79.2%, m.p. 135–136 °C. IR (KBr) *ν*_max_: 3412.5, 3232.6, 3094.4, 1621.5, 1574.5, 1483.6, 1384.1, 1286.7, 1192.1, 1082.7 cm^−1. 1^H NMR (300 MHz, DMSO-*d_6_*) δ: 2.16 (s, 3H, CH_3_), 7.33 (d, *J* = 3.69 Hz, 1H, FuH), 7.39 (d, *J* = 3.69 Hz, 1H, FuH), 7.41–7.54 (m, 2H, 2ArH–Fu), 7.61–7.64 (m, 1H, ArH–Fu), 8.21–8.24 (m, 1H, ArH–Fu), 10.61 (s, 1H, NH), 10.66 (s, 1H, NH). MS/ESI: *m*/*e* (%) 279.2 [M + H]^+^. Anal. Calcd. (%) for C_13_H_11_ClN_2_O_3_: C, 56.03; H, 3.98; N, 10.05. Found: C, 56.26; H, 4.07; N, 9.94.

***N*-Acetyl-*N*′-[5-(2′-fluorophenyl)-2-furoyl]hydrazine (III-4-1).** White solid: yield 74.6%, m.p. 150–151 °C. IR (KBr) *ν*_max_: 3415.2, 3263.1, 2994.8, 1643.8, 1523.5, 1452.6, 1323.1, 1261.7, 1153.1, 1123.6, 1093.4 cm^−1. 1^H NMR (300 MHz, DMSO-*d_6_*) δ: 2.16 (s, 3H, CH_3_), 6.97 (d, *J* = 3.66 Hz, 1H, FuH), 7.41 (d, *J* = 3.69 Hz, 1H, FuH), 7.69 (td, *J* = 7.77, 1.38 Hz, 1H, ArH–Fu), 7.83 (td, *J* = 7.67, 1.22 Hz, 1H, ArH–Fu), 8.00–8.05 (m, 2H, ArH–Fu), 10.44 (s, 1H, NH), 10.68 (s, 1H, NH). MS/ESI: *m*/*e* (%) 262.9 [M + H]^+^. Anal. Calcd. (%) for C_13_H_11_FN_2_O_3_: C, 59.54; H, 4.23; N, 10.68. Found: C, 59.63; H, 4.01; N, 10.83.

***N*-Acetyl-*N*′-[5-(3′-fluorophenyl)-2-furoyl]hydrazine (III-5-1)**. White solid: yield 69.5%, m.p. 143–144 °C. IR (KBr) *ν*_max_: 3398.9, 3123.6, 2978.1, 1653.5, 1583.5, 1455.6, 1354.5, 1233.4, 1154.1, 1083.5 cm^−1. 1^H NMR (300 MHz, DMSO-*d_6_*) δ: 2.16 (s, 3H, CH_3_), 7.38 (d, *J* = 3.66 Hz, 1H, FuH), 7.47 (d, *J* = 3.66 Hz, 1H, FuH), 7.80–7.83 (m, 1H, ArH–Fu), 8.21–8.25 (m, 1H, ArH–Fu), 8.39–8.42 (m, 1H, ArH–Fu), 8.83–8.86 (m, 1H, ArH–Fu), 10.65 (s, 1H, NH), 10.80 (s, 1H, NH). MS/ESI: *m*/*e* (%) 285.1 [M + Na]^+^. Anal. Calcd. (%) for C_13_H_11_FN_2_O_3_: C, 59.54; H, 4.23; N, 10.68. Found: C, 59.71; H, 4.48; N, 10.75.

***N*-Acetyl-*N*′-[5-(4′-fluorophenyl)-2-furoyl]hydrazine (III-6-1).** White solid: yield 80.1%, m.p. 161–162 °C. IR (KBr) *ν*_max_: 3404.2, 3232.3, 2883.4, 1643.5, 1546.3, 1463.2, 1362.1, 1223.3, 1153.8, 1062.1 cm^−1. 1^H NMR (300 MHz, DMSO-*d_6_*) δ: 2.16 (s, 3H, CH_3_), 7.41 (d, *J* = 3.69 Hz, 1H, FuH), 7.50 (d, *J* = 3.66 Hz, 1H, FuH), 8.24 (d, *J* = 7.05 Hz, 2H, ArH–Fu), 8.33–8.38 (m, 2H, ArH–Fu), 10.67 (s, 1H, NH), 10.77 (s, 1H, NH). MS/ESI: *m*/*e* (%) 263.1 [M + H]^+^. Anal. Calcd. (%) for C_13_H_11_FN_2_O_3_: C, 59.54; H, 4.23; N, 10.68. Found: C, 59.36; H, 3.99; N, 10.43.

***N*-Acetyl-*N*′-[5-(2′,4′-difluorophenyl)-2-furoyl]hydrazine (III-7-1).** White solid: yield 79.2%, m.p. 152–153 °C. IR (KBr) *ν*_max_: 3434.5, 3223.6, 3053.3, 1653.2, 1554.3, 1443.3, 1353.1, 1223.7, 1034.2 cm^−1. 1^H NMR (300 MHz, DMSO-*d_6_*) δ: 2.16 (s, 3H, CH_3_), 7.02 (t, *J* = 3.71 Hz, 1H, FuH), 7.35−7.49 (m, 4H, 1FuH + 3ArH–Fu), 8.19–8.25 (m, 1H, ArH–Fu), 10.65 (s, 1H, NH), 10.68 (s, 1H, NH). MS/ESI: *m*/*e* (%) 281.2 [M + H]^+^. Anal. Calcd. (%) for C_13_H_10_F_2_N_2_O_3_: C, 55.72; H, 3.60; N, 10.00. Found: C, 55.57; H, 3.68; N, 10.26.

***N*-acetyl-*N*′-[5-(2′,6′-difluorophenyl)-2-furoyl]hydrazine (III-8-1).** White solid: yield 79.8%, m.p. 139–140 °C. IR (KBr) *ν*_max_: 3411.8, 3072.4, 1643.5, 1565.4, 1472.6, 1332.1, 1262.3, 1171.9, 1033.5 cm^−1. 1^H NMR (300 MHz, DMSO-*d_6_*) δ: 2.16 (s, 3H, CH_3_), 7.23–7.29 (m, 2H, FuH + ArH–Fu), 7.35 (d, *J* = 3.63 Hz, 1H, FuH), 7.48–7.52 (m, 1H, ArH–Fu), 7.81–7.84 (m, 1H, ArH–Fu), 7.91–7.94 (m, 1H, ArH–Fu), 10.41 (s, 1H, NH), 10.59 (s, 1H, NH). MS/ESI: *m*/*e* (%) 280.9 [M + H]^+^. Anal. Calcd. (%) for C_13_H_10_F_2_N_2_O_3_: C, 55.72; H, 3.60; N, 10.00. Found: C, 55.98; H, 3.51; N, 9.87.

***N*-Acetyl-*N*′-[5-(2′-nitrophenyl)-2-furoyl]hydrazine (III-9-1).** Yellow solid: yield 82.3%, m.p. 138–139 °C. IR (KBr) *ν*_max_: 3417.4, 3223.5, 3063.1, 1663.7, 1553.4, 1434.1, 1273.7, 1145.9, 1073.4 cm^−1. 1^H NMR (300 MHz, DMSO-*d_6_*) δ: 2.16 (s, 3H, CH_3_), 7.16 (d, *J* = 3.57 Hz, 1H, FuH), 7.33–7.39 (m, 3H, FuH + 2ArH–Fu), 8.05 (d, *J* = 8.79 Hz, 2H, ArH–Fu), 10.43 (s, 1H, NH), 10.69 (s, 1H, NH). MS/ESI: *m*/*e* (%) 312.2 [M + Na]^+^. Anal. Calcd. (%) for C_13_H_11_N_3_O_5_: C, 53.98; H, 3.83; N, 14.53. Found: C, 53.71; H, 4.00; N, 14.73.

***N*-Acetyl-*N*′-[5-(3′-nitrophenyl)-2-furoyl]hydrazine (III-10-1).** Yellow solid: yield 81.4%, m.p. 143–144 °C. IR (KBr) *ν*_max_: 3402.1, 3227.4, 2799.5 1634.1, 1562.3, 1454.5, 1345.3, 1273.7, 1164.4 cm^−1. 1^H NMR (300 MHz, DMSO-*d_6_*) δ: 2.16 (s, 3H, CH_3_), 6.99 (t, *J* = 3.74 Hz, 1H, FuH), 7.29–7.38 (m, 2H, FuH + ArH–Fu), 7.45–7.53 (m, 1H, ArH–Fu), 8.24–8.32 (m, 1H, ArH–Fu), 10.66 (s, 1H, NH), 10.70 (s, 1H, NH). MS/ESI: *m*/*e* (%) 290.1 [M + H]^+^. Anal. Calcd. (%) for C_13_H_11_N_3_O_5_: C, 53.98; H, 3.83; N, 14.53. Found: C, 54.16; H, 3.65; N, 14.32.

***N*-Acetyl-*N*′-[5-(4′-nitrophenyl)-2-furoyl]hydrazine (III-11-1).** Yellow solid: yield 84.5%, m.p. 140–141 °C. IR (KBr) *ν*_max_: 3346.3, 3023.4, 1682.5, 1543.7, 1483.3, 1334.8, 1264.5, 1145.4, 1043.8 cm^−1. 1^H NMR (300 MHz, DMSO-*d_6_*) δ: 2.16 (s, 3H, CH_3_), 7.04–7.06 (m, 1H, ArH–Fu), 7.28–7.33 (m, 2H, FuH + ArH–Fu), 7.47 (d, *J* = 3.66 Hz, 1H, FuH), 7.54–7.59 (m, 1H, ArH–Fu), 10.52 (s, 1H, NH), 10.62 (s, 1H, NH). MS/ESI: *m*/*e* (%) 290.1 [M + H]^+^. Anal. Calcd. (%) for C_13_H_11_N_3_O_5_: C, 53.98; H, 3.83; N, 14.53. Found: C, 54.09; H, 4.11; N, 14.28.

***N*-Acetyl-*N*′-(5-phenyl-2-furoyl)hydrazine (III-12-1).** White solid: yield 75.6%, m.p. 129–130 °C. IR (KBr) *ν*_max_: 3402.2, 3223.5, 3057.3, 1643.5, 1563.8, 1464.3, 1352.2, 1253.9, 1172.1, 1034.5 cm^−1. 1^H NMR (300 MHz, DMSO-*d_6_*) δ: 2.16 (s, 3H, CH_3_), 7.19 (d, *J* = 3.60 Hz, 1H, FuH), 7.35 (d, *J* = 3.63 Hz, 1H, FuH), 7.37–7.43 (m, 1H, ArH–Fu), 7.47–7.52 (m, 2H, ArH–Fu), 7.97–8.00 (m, 2H, ArH–Fu), 10.62 (s, 1H, NH), 10.64 (s, 1H, NH). MS/ESI: *m*/*e* (%) 245.1 [M + H]^+^. Anal. Calcd. (%) for C_13_H_12_N_2_O_3_: C, 63.93; H, 4.95; N, 11.47. Found: C, 64.06; H, 5.17; N, 11.28.

***N*-Acetyl-*N*′-[5-(4′-methylphenyl)-2-furoyl]hydrazine (III-13-1).** White solid: yield 73.5%, m.p. 142–143 °C. IR (KBr) *ν*_max_: 3423.3, 3253.3, 2875.8, 1643.5, 1583.4, 1464.3, 1274.5, 1175.1, 1043.2 cm^−1. 1^H NMR (300 MHz, DMSO-*d_6_*) δ: 2.16 (s, 3H, CH_3_), 2.35 (s, 3H, CH_3_), 7.10 (d, *J* = 3.60 Hz, 1H, FuH), 7.27–7.33 (m, 3H, FuH + 2ArH–Fu), 7.85–7.88 (m, 2H, ArH–Fu), 10.57 (s, 1H, NH), 10.62 (s, 1H, NH). MS/ESI: *m*/*e* (%) 281.1 [M + Na]^+^. Anal. Calcd. (%) for C_14_H_14_N_2_O_3_: C, 65.11; H, 5.46; N, 10.85. Found: C, 65.25; H, 5.71; N, 10.64.

***N*-Acetyl-*N*′-[5-(4′-methoxyphenyl)-2-furoyl]hydrazine (III-14-1).** White solid: yield 79.2%, m.p. 137–138 °C. IR (KBr) *ν*_max_: 3402.3, 3243.2, 3012.2, 1635.8, 1574.5, 1454.4, 1383.1, 1289.5, 1216.7, 1142.1, 1064.5 cm^−1. 1^H NMR (300 MHz, DMSO-*d_6_*) δ: 2.16 (s, 3H, CH_3_), 3.82 (s, 3H, OCH_3_), 7.01–7.07 (m, 3H, FuH + 2ArH–Fu), 7.30 (d, *J* = 3.60 Hz, 1H, FuH), 7.90–7.93 (m, 2H, ArH–Fu), 10.43 (s, 1H, NH), 10.49 (s, 1H, NH). MS/ESI: *m*/*e* (%) 275.1 [M + H]^+^. Anal. Calcd. (%) for C_14_H_14_N_2_O_4_: C, 61.31; H, 5.14; N, 10.21. Found: C, 61.11; H, 5.13; N, 10.04.

***N*-Acetyl-*N*′-[5-(4′-bromophenyl)-2-furoyl]hydrazine (III-15-1).** White solid: yield 84.3%, m.p. 160–161 °C. IR (KBr) *ν*_max_: 3432.5, 3074.3, 1645.4, 1584.5, 1521.7, 1463.6, 1365.1, 1212.4, 1175.1, 1032.9 cm^−1. 1^H NMR (300 MHz, DMSO-*d_6_*) δ: 2.15 (s, 3H, CH_3_), 7.24 (d, *J* = 3.63 Hz, 1H, FuH), 7.34 (d, *J* = 3.63 Hz, 1H, FuH), 7.69–7.72 (m, 2H, ArH–Fu), 7.94–7.97 (m, 2H, ArH–Fu), 10.45 (s, 1H, NH), 10.73 (s, 1H, NH). MS/ESI: *m*/*e* (%) 345.1 [M + Na]^+^. Anal. Calcd. (%) for C_13_H_11_BrN_2_O_3_: C, 48.32; H, 3.43; N, 8.67. Found: C, 48.57; H, 3.28; N, 8.88.

***N*-Propionyl-*N*′-[5-(4′-cholorophenyl)-2-furoyl]hydrazine (III-3-2).** White solid: yield 81.6%, m.p. 110–111 °C. IR (KBr) *ν*_max_: 3418.1, 3213.5, 1691.3, 1632.1, 1591.2, 1520.3, 1481.3, 1280.6, 1211.3, 1121.4 cm^−1. 1^H NMR (300 MHz, DMSO-*d_6_*) δ: 1.12 (t, *J* = 6.96 Hz, 3H, CH_3_–C), 2.13 (q, *J* = 6.96 Hz, 2H, CO–CH_2_–C), 7.35 (d, *J* = 3.69 Hz, 1H, FuH),7.40 (d, *J* = 3.72 Hz, 1H, FuH), 7.42–7.54 (m, 2H, ArH–Fu), 7.61–7.64 (m, 1H, ArH–Fu), 8.25–8.28 (m, 1H, ArH–Fu), 10.48 (s, 1H, NH), 10.78 (s, 1H, NH). MS/ESI: *m*/*e* (%) 293.1 [M + H]^+^. Anal. Calcd. (%) for C_14_H_13_ClN_2_O_3_: C, 57.44; H, 4.48; N, 9.57. Found: C, 57.61; H, 4.28; N, 9.72.

***N*-Butyryl-*N*′-[5-(4′-cholorophenyl)-2-furoyl]hydrazine (III-3-3).** White solid: yield 78.3%, m.p. 121–122 °C. IR (KBr) *ν*_max_: 3415.6, 3245.1, 2934.3, 1641.6, 1604.5, 1551.4, 1478.5, 1286.5, 1253.6, 1146.2, 1173.6, 1015.7 cm^−1. 1^H NMR (300 MHz, DMSO-*d_6_*) δ: 0.96 (t, *J* = 7.50 Hz, 3H, CH_3_–C), 1.59–1.63 (m, 2H, C–CH_2_–C), 2.19 (t, *J* = 7.56 Hz, 2H, C–CH_2_–CO), 7.33 (d, *J* = 3.66Hz, 1H, FuH), 7.38 (d, *J* = 3.66 Hz, 1H, FuH), 7.41–7.54 (m, 2H, ArH–Fu), 7.60–7.63 (m, 1H, ArH–Fu), 8.22–8.25 (m, 1H, ArH–Fu), 10.32 (s, 1H, NH), 10.51 (s, 1H, NH). MS/ESI: *m*/*e* (%) 329.1 [M + Na]^+^. Anal. Calcd. (%) for C_15_H_15_ClN_2_O_3_: C, 58.73; H, 4.93; N, 9.13. Found: C, 58.52; H, 5.06; N, 9.31.

***N*-Isobutyryl-*N*′-[5-(4′-cholorophenyl)-2-furoyl]hydrazine (III-3-4).** White solid: yield 82.0%, m.p. 128–129 °C. IR (KBr) *ν*_max_: 3418.4, 3250.3, 1681.6, 1646.7, 1592.4, 1523.5, 1493.1, 1253.5, 1181.2, 1110.7 cm^−1. 1^H NMR (300 MHz, DMSO-*d_6_*) δ: 0.86 (d, 6H, *J* = 7.02 Hz, (CH_3_)_2_C), 2.11–2.14 (m, 1H, CO–CH–C), 7.33 (d, *J* = 3.69 Hz, 1H, FuH), 7.38 (d, *J* = 3.69 Hz, 1H, FuH), 7.41–7.54 (m, 2H, ArH–Fu), 7.61–7.64 (m, 1H, ArH–Fu), 8.22–8.25 (m, 1H, ArH–Fu), 10.34 (s, 1H, NH), 10.58 (s, 1H, NH). MS/ESI: *m*/*e* (%) 307.2 [M + H]^+^. Anal. Calcd. (%) for C_15_H_15_ClN_2_O_3_: C, 58.73; H, 4.93; N, 9.13. Found: C, 58.90; H, 4.81; N, 9.29.

***N*-Hexanoyl-*N*′-[5-(4′-cholorophenyl)-2-furoyl]hydrazine (III-3-5).** White solid: yield 78.0%, m.p. 140–141 °C. IR (KBr) *ν*_max_: 3399.4, 3213.6, 2887.4, 1621.4, 1591.6, 1534.7, 1491.5, 1474.3, 1271.8, 1111.5 cm^−1. 1^H NMR (300 MHz, DMSO-*d_6_*) δ: 0.94 (t, *J* = 7.52 Hz, 3H, CH_3_), 1.32–1.35 (m, 2H, CH_2_), 1.64–1.67 (m, 2H, CH_2_), 2.09–2.12 (m, 2H, CH_2_), 2.20 (t, *J* = 7.8 Hz, 2H, C–CH_2_–CO), 7.34 (d, *J* = 3.69 Hz, 1H, FuH), 7.39 (d, *J* = 3.69 Hz, 1H, FuH), 7.42–7.54 (m, 2H, ArH–Fu), 7.60–7.63 (m, 1H, ArH–Fu), 8.21–8.24 (m, 1H, ArH–Fu), 10.64 (s, 1H, NH), 10.66 (s, 1H, NH). MS/ESI: *m*/*e* (%) 335.1 [M + H]^+^. Anal. Calcd. (%) for C_17_H_19_ClN_2_O_3_: C, 60.99; H, 5.72; N, 8.37. Found: C, 61.13; H, 5.84; N, 8.45.

***N*-Heptanoyl-*N*′-[5-(4′-cholorophenyl)-2-furoyl]hydrazine (III-3-6).** White solid: yield 80.5%, m.p. 143–144 °C. IR (KBr) *ν*_max_: 3416.9, 3221.2, 2884.1, 1632.1, 1572.1, 1524.7, 1484.3, 1254.8, 1233.5, 1105.3 cm^−1. 1^H NMR (300 MHz, DMSO-*d_6_*) δ: 0.94 (t, 3H, *J* = 7.50 Hz, CH_3_), 1.24–1.29 (m, 6H, 3CH_2_), 1.54–1.57 (m, 2H, CH_2_), 2.15–2.17 (m, 2H, C–CH_2_–CO), 7.34 (d, *J* = 3.69 Hz, 1H, FuH), 7.39 (d, *J* = 3.69 Hz, 1H, FuH), 7.42–7.55 (m, 2H, ArH–Fu), 7.60–7.63 (m, 1H, ArH–Fu), 8.22–8.25 (m, 1H, ArH–Fu), 10.55 (s, 1H, NH), 10.68 (s, 1H, NH). MS/ESI: *m*/*e* (%) 349.1 [M + H]^+^. Anal. Calcd. (%) for C_18_H_21_ClN_2_O_3_: C, 61.98; H, 6.07; N, 8.03. Found: C, 62.16; H, 6.18; N, 7.93.

***N*-Octanoyl-*N*′-[5-(4′-cholorophenyl)-2-furoyl]hydrazine (III-3-7).** White solid: yield 82.5%, m.p. 149–150 °C. IR (KBr) *ν*_max_: 3398.5, 3114.7, 1607.5, 1492.5, 1451.7, 1250.6, 1221.1 cm^−1. 1^H NMR (300 MHz, DMSO-*d_6_*) δ: 0.96 (t, 3H, *J* = 7.50 Hz, CH_3_), 1.26–1.30 (m, 8H, 4CH_2_), 1.56–1.59 (m, 2H, CH_2_), 2.17–2.19 (m, 2H, C–CH_2_–CO), 7.34 (d, *J* = 3.69 Hz, 1H, FuH), 7.39 (d, *J* = 3.69 Hz, 1H, FuH), 7.42–7.55 (m, 2H, ArH–Fu), 7.60–7.63 (m, 1H, ArH–Fu), 8.22–8.25 (m, 1H, ArH–Fu), 10.53 (s, 1H, NH), 10.64 (s, 1H, NH). MS/ESI: *m*/*e* (%) 363.2 [M + H]^+^. Anal. Calcd. (%) for C_19_H_23_ClN_2_O_3_: C, 62.89; H, 6.39; N, 7.72. Found: C, 63.06; H, 6.25; N, 7.60.

***N*-Nonanoyl-*N*′-[5-(4′-cholorophenyl)-2-furoyl]hydrazine (III-3-8).** White solid: yield 80.8%, m.p. 159–160 °C. IR (KBr) *ν*_max_: 3410.4, 3206.7, 2931.3, 1620.5, 1617.5, 1532.6, 1484.6, 1369.4, 1289.3, 1110.3 cm^−1. 1^H NMR (300 MHz, DMSO-*d_6_*) δ: 0.93 (t, *J* = 7.50 Hz, 3H, CH_3_), 1.28–1.33 (m, 10H, 5CH_2_), 1.58–1.60 (m, 2H, CH_2_), 2.21 (t, 2H, *J* = 7.50 Hz,), 7.33 (d, *J* = 3.66 Hz, 1H, FuH), 7.39 (d, *J* = 3.69 Hz, 1H, FuH), 7.41–7.54 (m, 2H, ArH–Fu), 7.60–7.63 (m, 1H, ArH–Fu), 8.22–8.25 (m, 1H, ArH–Fu), 10.47 (s, 1H, NH), 10.62 (s, 1H, NH). MS/ESI: *m*/*e* (%) 377.3 [M + H] ^+^. Anal. Calcd. (%) for C_20_H_25_ClN_2_O_3_: C, 63.74; H, 6.69; N, 7.43. Found: C, 63.58; H, 6.83; N, 7.27.

***N*-Decanoyl-*N*′-[5-(4′-cholorophenyl)-2-furoyl]hydrazine (III-3-9).** White solid: yield 78.2%, m.p. 162–163 °C. IR (KBr) *ν*_max_: 3397.1, 3212.4, 2962.4, 1631.5, 1527.3, 1478.9, 1295.7 cm^−1. 1^H NMR (300 MHz, DMSO-*d_6_*) δ: 0.90 (t, 3H, *J* = 6.5 Hz, H-9), 1.27–1.32 (m, 12H, 5CH_2_), 1.59–1.61 (m, 2H, CH_2_), 2.19–2.21 (m, 2H, C–CH_2_–CO), 7.33 (d, *J* = 3.66 Hz, 1H, FuH), 7.39 (d, *J* = 3.69 Hz, 1H, FuH), 7.41–7.54 (m, 2H, ArH–Fu), 7.60–7.63 (m, 1H, ArH–Fu), 8.22–8.25 (m, 1H, ArH–Fu), 10.46 (s, 1H, NH), 10.60 (s, 1H, NH). MS/ESI: *m*/*e* (%) 413.2 [M + Na]^+^. Anal. Calcd. (%) for C_21_H_27_ClN_2_O_3_: C, 64.52; H, 6.96; N, 7.17. Found: C, 64.71; H, 7.10; N, 6.99.

### 3.3. Crystallography

Compound **III-3-2** was recrystallized from methanol to give colorless crystals suitable for X-ray single crystal diffraction. Cell constants at 113(2) K were determined by a least-square fit to the setting parameters of independent reflections measured on a Bruker SMART [12] 1000 CCD area-detector diffractometer (Bruker, Fallanden, Switzerland) with graphite-monochromated Mo K_α_ radiation (λ *=* 0.071070 nm) and operating in the phi and scan modes. The structure was solved by the direct method with SHELXS-97 [24–27] and refined by the full-matrix least squares method on F2 data using SHELXL-97 [27,28]. The empirical absorption corrections were applied to all intensity data. H atom of N–H was initially located in a different Fourier map and was refined with the restraint *U*_iso_ (H) = 1.2 *U*_eq_ (N). Other H atoms were positioned geometrically and refined using a riding model, with *d* (C···H) = 0.093–0.097 nm and *U*_iso_ (H) = 1.2 *U*_eq_ (C) or 1.5 *U*_eq_ (C–methyl). The crystal data in CIF format have been deposited at the Cambridge Crystallographic Data Centre with deposition number CCDC 935116.

### 3.4. Bioassay

#### 3.4.1. Insecticidal Activity

Assessments were made on a dead/live basis. Evaluations are based on a percentage scale of 0–100, which 0 equals no activity and 100 equals total kill. The bioassay was repeated three times and the result of bioactivity was the average of these replicates. Error in the experiments was 5%. The commercialized insecticide RH-5849 and an aromatic derivative (Scheme 1, **VI-1**: R^1^ = 4-CH_3_, R^2^ = 4-Cl) were tested as controls under the same conditions. EXCEL2007 was applied to analyze bioassay data.

Larvicidal activity against *Culex pipiens pallens*. The larvicidal activity was evaluated at the preliminary test concentration of 10 mg·L^−1^ against the fourth-instar *Culex pipiens pallens* by the water immersion method under conditions of 27 ± 2 °C, photoperiod of 10:14 (light:dark), and relative humidity 50%–70%. All the test beakers containing twenty *Culex pipiens pallens* were evaluated 8 days after treatment.

Larvicidal activity against the oriental armyworm (*Mythimna separata*). The larvicidal activity of the title compounds against the oriental armyworm was evaluated by foliar application. For the foliar armyworm tests, individual corn leaves were placed on moistened pieces of filter paper in Petri dishes. The leaves were then sprayed with the test solution at the preliminary test concentration of 200 mg·L^−1^ and allowed to dry. The dishes were infested with 10 fourth-instar oriental armyworm larvae. Percentage mortalities were evaluated 4 days after treatment.

Larvicidal activity against the diamondback moth (*Plutella xylostella*). The larvicidal activity of the title compounds against the diamondback moth was tested by the leaf-dip method. Leaf disks (1.8 cm diameter) were cut from fresh cabbage leaves and then dipped in the test solution for 15 s at the preliminary test concentration of 200 mg·L^−1^. After air-drying, the treated leaf disks were placed in a Petri dish (9 cm diameter), lined with a piece of filter paper and then 10 second-instar diamondback moth larvae were transferred to the Petri dish. Percentage mortalities were evaluated 6 days after treatment.

#### 3.4.2. Fungicidal Activity

Using pot culture tests according to references [12,26], the *in vivo* fungicidal activities of the title compounds against *B. cinerea*, *C. cassiicola*, *F. oxysporum*, and *R. solanii* were evaluated in a greenhouse with four commercial fungicides, 40% pyrimethanil SC, 75% chlorothalonil WP, 40% flusilazole EC, and 3% validamycin AS and an aromatic derivative (Scheme 1, **VI-1**: R^1^ = 4-CH_3_, R^2^ = 4-Cl) as controls. All the strains were conserved at the Institute of Plant Protection, Chinese Academy of Agricultural Science, Beijing, China. *B. cinerea*, *C. cassiicola*, *F. oxysporum*, and *R. solanii* were maintained on potato dextrose agar (PDA) medium at 4 °C. The culture plates were cultivated at 24 ± 1 °C. Germination was conducted by soaking cucumber seeds in water for 2 h at 50 °C and then keeping the seeds moist for 24 h at 28 °C in an incubator. When the radicles were 0.5 cm, the seeds were grown in plastic pots containing a 1:1 (*v*/*v*) mixture of vermiculite and peat. Cucumber plants used for inoculations were at the stage of two seed leaves.

Tested compounds and commercial fungicides were sprayed with a hand spray onto the surface of the seed leaves and were used for *B. cinerea*, *C. cassiicola*, and *R. solanii* at the standard concentration of 500 μg·mL^−1^. Tested compounds and commercial fungicide were applied to the cucumber plants by the irrigating method and used for *F. oxysporum.* Three replicates were used per treatment.

After drying, inoculation of *C. cassiicola* was carried out by spraying a conidial suspension, *F. oxysporum* was innoculated by embryo root inoculation, while inoculations of *B. cinerea* and *R. solanii* were carried out by spraying mycelial suspensions. Three replicates were performed. After inoculation, the plants were maintained at 24 ± 1 °C and above 80% relative humidity.

The fungicidal activity was evaluated when the untreated cucumber plant (blank control) fully developed symptoms. The area of inoculated treated leaves covered by disease symptoms was assessed and compared to that of untreated ones to determine the average disease index. The relative control efficacy of compounds compared to the blank assay was calculated via Equation (1):

(1) I(%)=[(CK-PT)/CK]×100%

where *I* is relative control efficacy, *CK* is the average disease index during the blank assay, and *PT* is the average disease index after treatment during testing.

#### 3.4.3. Anti-Tumor Activity

All the title compounds were dissolved in DMSO and screened for preliminary anti-tumor activity against four different cell lines: a human promyelocytic leukemic cell line (HL-60), a human hepatocellular carcinoma cell line (Bel-7402), a human gastric carcinoma cell line (BGC-823), and a human nasopharyngeal carcinoma cell line (KB) at a concentration gradient of 0.1, 1.0, 10, 50, and 100 μM. The commercial drug doxorubicin and an aromatic derivative (Scheme 1, **VI-1**: R^1^ = 4-CH_3_, R^2^ = 4-Cl) were used as controls in the bioassay. Three replicates were performed.

The four types of cell line were grown and maintained in RPMI-1640 medium supplemented with 10% fetal bovine serum (FBS), penicillin (100 U·mL^−1^), and streptomycin (100 μg·mL^−1^) at 37 °C in humidified incubators in an atmosphere of 5% CO_2_.

All the experiments were performed on exponentially growing cancer cells. Numbers of viable cancer cells were determined by MTT [3-(4,5-dimethylthiazol-2-yl)-2,5-diphenyl tetrazoliumbromide] [27] and SRB (sulforhodamine B) [28] assays. The cancer cells ((1–2.5) × 10^4^ cells·mL^−1^) were inoculated in 96-well culture plates (180 μL/well). After 24 h, 20 μL of culture medium containing compounds of various concentrations were added to the wells and the cells were incubated for 48 h. to control cells, 20 μL of RPMI-1640 medium was added. HL-60 cells were assayed by MTT, whereas Bel-7402, BGC-823, and KB cells were assayed by SRB. The absorbance of each well was measured using a microculture plate reader at 570 nm (MTT) and 540 nm (SRB), respectively. The inhibition rate was calculated according to Equation (2):

(2) $$\text{Inhibition rate}=(OD_{\text{control}}-OD_{\text{treated}})/OD_{\text{control}}\times 100\%$$

## 4. Conclusions

In summary, a series of novel 5-substituted-2-furoyl diacylhydazide derivatives with aliphatic chain **III** was designed and synthesized. Their anti-tumor and anti-fungal tests indicated that most of the title compounds showed significant bioactivity against the tested tumor cell lines and fungi. The bioactivity was improved and better than that of the aromatic diacylhydrazides. The aliphatic chain length and the structure and position of the R^2^ group had a significant impact on the bioactivities. Generally, the shorter the chain, the better the corresponding activity was. When R^2^ was an electron-withdrawing group, such as NO_2_ and Cl at the *ortho* or *para* position, the compounds exhibited higher activity. While the electron-donating groups at the *para* or *meta* position on the phenyl ring decreased the activity. The title compounds with aliphatic chains exhibited moderate or marginal insecticidal activity, which was neither improved nor decreased compared to that of the aromatic diacylhydrazides.

## Figures and Tables

**Table 1.** Crystal and experimental data of compound **III-3-2**.

Empirical formula	C_14_H_13_ClN_2_O_3_
Formula weight	292.71
*T*	113(2) K
Wavelength	0.71070 Å
Crystal system	Orthorhombic
Space group	C 2 2 21
Unit cell dimensions	*a* = 8.4488(4) Å, α = 90° *b* = 20.1074(13) Å, β = 90° *c* = 16.1363(10) Å, γ = 90°
Volume	2741.3(3) Å^3^
*Z*	8
*D_x_*	1.418 mg·m^−3^
Absorption coefficient	0.287 mm^−1^
*F* (0 0 0)	1216
Crystal dimensions	0.22 × 0.20 × 0.20 mm
θ range for data collection	2.03 to 27.85
Completeness to θ = 27.85	99.9%
Limiting indices	−11 ≤ *h* ≤ 10, −26 ≤ *k* ≤ 23,−21 ≤ *l* ≤ 21
Reflection collected/unique	12970/3269 [*R*(int) = 0.0486]
Absorption correction	Semi-empirical from equivalents
Max. and min. transmission	0.9448 and 0.9395
Data/restraints/parameters	3269/0/184
Goodness-of-fit on *F*^2^	1.101
Final *R* indices [*I* > 2σ (*I*)]	*R*_1_ = 0.0693, w*R*_2_ = 0.1949
2θ_max_	55.7° with Mo *K*_α_
(Δρ)_max_	1.204 eÅ^−3^
(Δρ)_min_	−0.406 eÅ^−3^
Program system	SHELXS-97, SHELXL-97
Structure determination	Direct method
Refinement	Full-matrix least-squares on *F*^2^
CCDC No.	935116

**Table 2.** Selected bond lengths, angels, and torsion angles of compound **III-3-2**.

Lengths	(Å)	Angles	(°)	Torsion angles	(°)
Cl(1)–C(1)	1.825(5)	C(11)–N(1)–N(2)	118.4(4)	C(11)–N(1)–N(2)–C(12)	102.3(5)
N(1)–C(11)	1.355(6)	C(12)–N(2)–N(1)	120.2(4)	C(6)–C(1)–C(2)–C(3)	2.3(7)
N(1)–N(2)	1.396(5)	C(10)–O(1)–C(7)	106.8(4)	Cl(1)–C(1)–C(2)–C(3)	−177.6(3)
N(2)–C(12)	1.343(6)	C(2)–C(1)–Cl(1)	119.1(4)	O(1)–C(7)–C(8)–C(9)	2.5(5)
O(1)–C(10)	1.364(5)	O(1)–C(7)–C(4)	117.2(4)	N(2)–N(1)–C(11)–O(2)	−1.6(7)
O(2)–C(11)	1.238(5)	O(2)–C(11)–N(1)	123.5(4)	N(2)–N(1)–C(11)–C(10)	176.9(4)
O(3)–C(12)	1.218(5)	O(3)–C(12)–N(2)	124.2(4)	O(1)–C(10)–C(11)–O(2)	171.7(3)
C(12)–C(13)	1.531(6)	N(2)–C(12)–C(13)	112.8(3)	N(1)–N(2)–C(12)–O(3)	5.8(7)
C(8)–C(9)	1.386(7)	C(14)–C(13)–C(12)	109.3(5)	N(1)–N(2)–C(12)–C(13)	−175.2(4)
C(9)–C(10)	1.368(7)	O(3)–C(12)–C(13)	123.0(4)	O(3)–C(12)–C(13)–C(14)	−70.8(6)
C(10)–C(11)	1.465(6)	C(6)–C(1)–C(2)	122.5(4)	N(2)–C(12)–C(13)–C(14)	110.2(5)
C(4)–C(7)	1.455(6)	O(1)–C(7)–C(8)	109.1(4)	C(10)–O(1)–C(7)–C(4)	177.6(3)

**Table 3.** The dihedral angles and the mean deviation of the planes in compound **III-3-2**.

Different planes	Dihedral angles (°)	Defination of the planes	The mean deviation of the plane (Å)
Plane I and plane II	10.0	Plane I (C1 to C6)	0.0153
Plane I and plane III	12.4	Plane II (C7 to C10, O1)	0.0150
Plane I and plane IV	56.9	Plane III (O2, C11, N1, N2)	0.0047
Plane II and plane III	6.8	Plane IV (O3, C12, C13, C14)	0.3708
Plane II and plane IV	47.7		
Plane III and plane IV	50.6		

**Table 4.** Larvicidal activity of title compounds **III**.

Compd.	R^1^	R^2^	*Plutella xylostella*	*Mythimna separata*	*Culex pipiens pallens*
		
			Larvicidal activity (%) at 200 mg·L^−1^	Larvicidal activity (%) at 200·mg L^−1^	Larvicidal activity (%) at 10 mg·L^−1^
**III-3-1**	CH_3_	4-Cl	45	30	20
**III-3-2**	CH_2_CH_3_	4-Cl	40	30	20
**III-3-3**	*n*-C_3_H_7_	4-Cl	55	40	30
**III-3-4**	*i*-C_3_H_7_	4-Cl	30	45	45
**III-3-5**	*n*-C_5_H_11_	4-Cl	35	30	20
**III-3-6**	*n*-C_6_H_13_	4-Cl	25	35	10
**III-3-7**	*n*-C_7_H_15_	4-Cl	30	30	10
**III-3-8**	*n*-C_8_H_17_	4-Cl	10	15	20
**III-3-9**	*n*-C_9_H_19_	4-Cl	15	10	30
**III-1-1**	CH_3_	2-Cl	45	35	40
**III-2-1**	CH_3_	3-Cl	20	20	10
**III-4-1**	CH_3_	2-F	35	30	45
**III-5-1**	CH_3_	3-F	10	10	20
**III-6-1**	CH_3_	4-F	35	30	40
**III-7-1**	CH_3_	2,4-di-F	40	30	30
**III-8-1**	CH_3_	2,6-di-F	60	50	60
**III-9-1**	CH_3_	2-NO_2_	25	20	30
**III-10-1**	CH_3_	3-NO_2_	35	10	20
**III-11-1**	CH_3_	4-NO_2_	20	30	20
**III-12-1**	CH_3_	H	10	35	35
**III-13-1**	CH_3_	4-CH_3_	35	30	25
**III-14-1**	CH_3_	4-OCH_3_	30	25	20
**III-15-1**	CH_3_	4-Br	10	20	25
**IV-1**	4-CH_3_	4-Cl	88	85	90
RH-5849			100	100	100

**Table 5.** Fungicidal activities of title compounds against four fungus species at 500 μg·mL^−1^ *in vivo.*

Compd.	R^1^	R^2^	Control efficacy (%)			

			*F. oxysporum*	*C. cassiicola*	*B. cinerea*	*R. solanii*
**III-3-1**	CH_3_	4-Cl	33.41 ± 1.22	19.32 ± 0.93	84.23 ± 2.43	93.43 ± 1.63
**III-3-2**	CH_2_CH_3_	4-Cl	34.23 ± 0.84	32.43 ± 1.23	61.23 ± 0.51	62.42 ± 1.61
**III-3-3**	*n*-C_3_H_7_	4-Cl	16.32 ± 1.12	41.32 ± 1.31	52.34 ± 1.23	51.32 ± 1.52
**III-3-4**	*i*-C_3_H_7_	4-Cl	15.76 ± 0.42	36.43 ± 1.49	55.13 ± 1.02	48.32 ± 0.81
**III-3-5**	*n*-C_5_H_11_	4-Cl	9.32 ± 0.65	12.43 ± 0.91	11.35 ± 0.80	43.32 ± 1.41
**III-3-6**	*n*-C_6_H_13_	4-Cl	33.62 ± 1.03	28.23 ± 0.54	27.53 ± 1.43	52.32 ± 0.97
**III-3-7**	*n*-C_7_H_15_	4-Cl	11.42 ± 0.52	12.61 ± 0.61	25.12 ± 0.91	32.21 ± 0.72
**III-3-8**	*n*-C_8_H_17_	4-Cl	21.34 ± 0.53	22.34 ± 0.72	30.52 ± 0.79	44.62 ± 0.72
**III-3-9**	*n*-C_9_H_19_	4-Cl	19.43 ± 0.63	12.33 ± 0.59	32.39 ± 0.53	52.52 ± 1.02
**III-1-1**	CH_3_	2-Cl	35.85 ± 0.55	28.24 ± 2.00	82.38 ± 3.02	84.13 ±1.16
**III-2-1**	CH_3_	3-Cl	25.73 ± 1.35	38.62 ± 1.45	33.54 ± 2.12	48.63 ± 1.63
**III-4-1**	CH_3_	2-F	18.61 ± 0.62	12.63 ± 1.27	62.36 ± 1.72	65.62 ± 1.82
**III-5-1**	CH_3_	3-F	9.32 ± 0.24	13.72 ± 0.85	35.43 ± 1.53	25.53 ± 1.63
**III-6-1**	CH_3_	4-F	18.52 ± 1.34	34.43 ± 1.62	48.45 ± 2.04	54.56 ± 1.23
**III-7-1**	CH_3_	2,4-di-F	9.53 ± 0.53	23.83 ± 1.11	63.32 ± 2.21	51.42 ± 1.51
**III-8-1**	CH_3_	2,6-di-F	13.21 ± 2.12	23.22 ± 1.42	36.13 ± 1.23	26.32 ± 1.26
**III-9-1**	CH_3_	2-NO_2_	62.34 ± 1.41	51.52 ± 2.34	82.62 ± 2.21	88.62 ± 1.62
**III-10-1**	CH_3_	3-NO_2_	47.34 ± 1.03	43.53 ± 1.34	37.43 ± 1.62	36.62 ± 1.63
**III-11-1**	CH_3_	4-NO_2_	34.62 ± 1.23	32.53 ± 1.72	92.52 ± 2.71	38.43 ± 0.53
**III-12-1**	CH_3_	H	32.61 ± 1.34	63.72 ± 1.23	66.62 ± 2.52	39.53 ± 2.32
**III-13-1**	CH_3_	4-CH_3_	12.82 ± 1.01	35.33 ± 1.73	27.63 ± 1.73	49.34 ± 1.35
**III-14-1**	CH_3_	4-OCH_3_	31.72 ± 1.72	28.92 ± 1.45	12.42 ± 1.43	52.54 ± 2.62
**III-15-1**	CH_3_	4-Br	40.42 ± 2.51	18.62 ± 0.82	32.23 ± 0.66	23.72 ± 1.32
**IV-1**	4-CH_3_	4-Cl	19.78 ± 0.84	48.56 ± 1.21	21.45 ± 0.97	20.78 ± 1.06
Acetone (blank control)			1.92 ± 0.82	2.25 ± 0.72	1.83 ± 0.53	2.62 ± 0.84
Fungicides #			94.17 ± 1.80 a	95.21 ± 1.94 b	89.57 ± 2.15 c	92.21 ± 2.41 d

# , Control fungicides;

a , 40% flusilazole EC;

b , 75% chlorothalonil WP;

c , 40% pyrimethanil SC; and

d , 3% validamycin AS.

Results are expressed as the mean ± SD (three experiments).

**Table 6.** Anti-tumor activity of title compounds **III**.

Compd.	R^1^	R^2^	IC_50_ values (μM)			

			HL-60	BGC-823	Bel-7402	KB
**III-3-1**	CH_3_	4-Cl	21.6	32.5	15.2	38.5
**III-3-2**	CH_2_CH_3_	4-Cl	35.8	25.9	56.7	32.1
**III-3-3**	*n*-C_3_H_7_	4-Cl	42.5	125.8	89.7	56.7
**III-3-4**	*i*-C_3_H_7_	4-Cl	45.8	56.8	56.9	198.4
**III-3-5**	*n*-C_5_H_11_	4-Cl	56.8	78.9	128.2	56.9
**III-3-6**	*n*-C_6_H_13_	4-Cl	65.4	198.4	98.7	95.7
**III-3-7**	*n*-C_7_H_15_	4-Cl	156.2	268.1	56.9	346.7
**III-3-8**	*n*-C_8_H_17_	4-Cl	286.5	96.8	389.8	156.8
**III-3-9**	*n*-C_9_H_19_	4-Cl	483.5	185.3	149.8	81.5
**III-1-1**	CH_3_	2-Cl	25.3	29.4	28.7	59.7
**III-2-1**	CH_3_	3-Cl	56.8	189.5	56.8	125.4
**III-4-1**	CH_3_	2-F	59.4	48.2	45.1	198.7
**III-5-1**	CH_3_	3-F	254.3	156.2	45.9	65.4
**III-6-1**	CH_3_	4-F	52.4	23.5	59.7	89.4
**III-7-1**	CH_3_	2,4-di-F	53.6	45.2	49.2	98.4
**III-8-1**	CH_3_	2,6-di-F	25.9	10.8	18.9	29.7
**III-9-1**	CH_3_	2-NO_2_	59.3	56.8	158.2	286.4
**III-10-1**	CH_3_	3-NO_2_	159.5	256.4	98.7	45.8
**III-11-1**	CH_3_	4-NO_2_	102.3	152.1	94.1	56.4
**III-12-1**	CH_3_	H	56.8	98.5	59.2	184.6
**III-13-1**	CH_3_	4-CH_3_	63.1	98.4	65.1	187.5
**III-14-1**	CH_3_	4-OCH_3_	45.5	158.4	108.9	204.2
**III-15-1**	CH_3_	4-Br	526.7	254.2	125.9	253.4
**IV-1**	4-CH_3_	4-Cl	598.7	421.8	261.5	398.7
doxorubicin			35.6	10.2	9.7	15.8
